# “I just very much love the journal”:  Understanding the community-led publishing landscape at the University of Cambridge

**DOI:** 10.12688/f1000research.161163.1

**Published:** 2025-03-05

**Authors:** Samuel Moore, Mandy Wigdorowitz

**Affiliations:** 1 Cambridge University Library, University of Cambridge, Cambridge, UK; 2 Theoretical and Applied Linguistics, University of Cambridge, Cambridge, UK; 3 Modern Languages and Classics, The University of Alabama, Tuscaloosa, Alabama, USA; 4 University of Johannesburg, Johannesburg, South Africa

**Keywords:** Cambridge, community-led publishing projects, open access, open research, publishing, scholarly communication

## Abstract

**Background:**

A growing movement of researcher-driven publishing projects has emerged in response to several challenges and shifts within the academic publishing landscape. One publishing initiative in this area are what we term community-led publishing projects (CPPs), which are produced entirely by academics, librarians and students without any involvement of the commercial publishing industry. CPPs are part of a growing global movement but their values and practices remain underexplored. This article presents findings on the landscape of CPPs at the University of Cambridge.

**Methods:**

A landscape analysis was undertaken to identify and describe the various CPPs at Cambridge. A subset of 10 journal editors were subsequently interviewed to explore the practices, motivations and needs of these initiatives.

**Results:**

Thirty-four CPPs were identified across a range of disciplines with a variety of publishing practices, open access status and professionalisation. From the interviews, CPPs were driven by an array of motivations including volunteers who are dedicated to their disciplines, who care for publishing, have a responsibility to disseminate their own research and who acknowledge the value these projects bring. They have complicated relationships with open access, being encouraged by public access to knowledge while maintaining a desire for print and being critical of some cultures of publishing brought on by the turn to openness. Practically, they employ a “DIY” approach due to the availability of resources but in doing so adhere to professional standards. Their success relies on collaboration and support, leveraging networks, technical and financial backing, and ensuring sustainability through careful handover.

**Conclusions:**

This study helps us better understand the scope and practices of community-led publishing at a research-intensive university in the UK. It shows that CPPs are valuable for a variety of reasons and that universities, funders and governments should support such projects to ensure the preservation of unique scholarly content.

## 1. Introduction

In recent years, academic publishing has been entirely transformed by open access (OA) forms of communication. Publishing is rapidly heading towards a situation in which the vast majority of research will be OA immediately upon publishing ( Piwowar, Priem, and Orr 2019). Much of this transition has been enabled by article processing charges (APCs) and, more recently, the replacement of journal subscriptions by transformative agreements that bundle together the cost of subscribing to back content and publishing new content OA. Yet this reorientation adds a pernicious dimension to publishing in that it encourages publishers to publish as many articles as they can in order to make the greatest returns possible. This turn to volume-based business models has led to sloppier publishing, lower human oversight and consequent issues of research integrity ( Hanson et al. 2023). In many ways, then, the commercial actors who control publishing have remained the same; they merely exploit the system in different ways.

As the publishing industry consolidates its power over research communication, it is important to acknowledge one important lineage of the OA movement: namely, community-led publishers organised entirely by academics, students and library workers. These community-led publishers were a key feature of the early Web, theorised elsewhere as the ‘pre-history’ of OA, and take a ‘do-it-yourself’ (‘DIY’) approach to publishing that purposefully eschews professional publishing houses ( Moore 2019). These publishers saw their work as an extension of their research, their activism or their scholarly commitment to their disciplines, often relying on a shoestring budget to do so. Community-led publishing has been an important part of OA publishing, and a flourishing ecosystem of community-led publishers exists globally today, although their hegemonic influence is somewhat limited by the complexity of the scholarly publishing landscape.

Nevertheless, community-led publishing has found momentum in the recent turn to ‘diamond’ forms of OA that do not charge fees to publish or read content ( Becerril, Arianna et al. 2021). Diamond OA journals do not exclude anyone on economic grounds and so are seen as one way of mitigating the impact of APCs and transformative agreements, although their long-term sustainability is a continual area of concern, not least due to their frequent over-reliance on volunteer labour in the absence of formal revenue-generating models. In problematising traditional methods of sustainability, diamond OA could allow us to work through the problems of commercial publishing by reinvigorating non-commercial approaches and finding forms of sustainability in alternative institutional arrangements, such as within the university. Diamond OA and community-led publishing are not synonymous, but there is a substantial overlap between DIY publishing projects and the kinds of publishers that fall under the diamond OA definition.

This article offers a snapshot of the community-led publishing landscape at the University of Cambridge, aiming to understand the community-led publishing projects (CPPs) that exist, how they publish their work and what their motivations are. We are interested in the kinds of practices that these CPPs engage in, why they do what they do, and how their work might be supported in the university and beyond. Through both a landscape overview and qualitative interviews with selected projects, we hope to provide an overview of the CPPs undertaken within a research-intensive university. This research will therefore contribute to the growing literature on alternative forms of publishing, particularly on how community-led approaches can offer a counterpoint to the commercial publishing landscape.

It is important to note from the outset that this study both is not and could not be a systematic overview of all the DIY publishing projects undertaken at Cambridge. We are terming our approach a ‘snapshot’ primarily to highlight the decisions taken around what to include and exclude, alongside the difficulties of identifying such publishing projects. While we identified over 30 CPPs, we are confident there are many more within the Cambridge community that we have missed, simply because the university is so decentralised and publishing projects are difficult to locate. This also raises the question of what constitutes a Cambridge-based CPP: Does the editor need to be based at Cambridge?; Does the publication need to be based within a Cambridge research institute or department?; How many of the journal’s editorial board members or authors need to be based at Cambridge? We deliberately avoided a firm answer to these questions by taking an expansive understanding of what constitutes a Cambridge-based CPP that took a number of these factors into account and allowed us to make a judgement call in each case. Furthermore, despite being a non-academic arm of the University of Cambridge, we were not analysing journals published by Cambridge University Press & Assessment, which is a professional, commercial publisher. We deliberately discounted any publications that were published under the name of a professional publishing house, although some of the journals use professional services for infrastructure or printing. We made a judgement call based on whether the journal seemed community-led, rather than professionally published, again opting for an inclusive approach to any grey areas.

### 1.1 Background

Historically speaking, scholarly communities have until recently required the services of professional printers and publishers to distribute their work. Publishing was originally organised by learned societies who needed to cover the costs of publication through benefactors and patrons, often allowing them to distribute their printed work for free ( Fyfe et al. 2017, 5). It wasn’t until the 1950s that publishing itself commercialised as a self-sustaining industry and not until the 1990s that it was marketised to the business that is recognisable today. This process of marketisation has meant that publishing has increasingly been taken out of the control of academic communities and is now governed by the market at large. An extreme example of this phenomenon is the rise of the APC, a payment by authors for the OA publication of their article. APC business models reward greater article volume, which has led to commercial publishers pressuring editorial boards into accepting increasing numbers of papers for maximum returns ( Fazackerley 2023).

While more mission-driven publishers such as learned societies and university presses still exist as important features of the publishing landscape, they too have been shaped by the drive for commercial outcomes and self-sustaining forms of revenue ( Adema 2022, 142). As universities withdraw the subsidies they previously provided, university presses are also forced to operate as businesses by breaking even through sales and other commercial returns, again ceding control to market logic. Learned societies now also face declining revenues that they previously put towards their scholarly activities, with those partnering with commercial publishers seemingly most affected ( Johnson and Malcolmson 2024). Commercial decision-making has profoundly shaped the ways in which academics publish their work.

Yet while professional forms of publishing are increasingly commercialised, there exists a flourishing ecosystem of community-led publishers operating within and across universities without recourse to professional publishing services but supported through self-organisation and a DIY approach to publishing their work. Enabled by the Web and digital technologies, this ecosystem is heterogenous in terms of subject matter, publication practices, financial support and many other features. Limited work has been undertaken to understand some of these publishers in a range of contexts. For example, Adema and Stone conducted a qualitative study of 14 ‘academic-led’ publishers, all of whom published OA material, ranging from DIY outfits to more professional publishers. Motivations included OA to knowledge, independence from publishing houses, publishing alternative or experimental content, and the desire to control their own work ( Adema, Stone, and Keene 2017, 43–47). These presses publish books, journals and born-digital projects with a high degree of heterogeneity of practice.

More recently, the turn to diamond OA has led researchers to analyse journals that rely less on commercial models and more on institutional forms of support, many of whom would be considered community-led in the sense we are describing. The Developing Institutional Open Access Publishing Models to Advance Scholarly Communication (DIAMAS) project aims to study these journals and devise forms of support for their activities. The project conducted a landscape study of institutional publishing activities (with a particular focus on the European research area), receiving 685 survey responses from 48 countries. The authors found that these publishers were highly influenced by their localities with significant differences between countries but fewer differences within them, such as languages, forms of institutional support and publishing practices ( Armengou et al. 2023a). In a separate report, the DIAMAS project found that many journals were supported institutionally and through in-kind support and volunteer labour, showing a range of different practices between the journals studied ( Brun, Pontille, and Torny 2024). The authors concluded that more must be done by universities to enable the diamond OA ecosystem, particularly through fixed and permanent forms of funding that allow them to undertake their work.

Other work has been undertaken on the community-led OA landscape within national contexts, such as Switzerland and Germany. The Germany study identified 345 distinct diamond OA journals, although offered no descriptive characteristics beyond raw numbers. The Swiss ‘Platinum Open Access Funding (PLATO)’ study identified 186 diamond OA journals in Switzerland, describing their work as follows:

Editorial tasks are mainly done by small teams of collaborators, often young researchers in the roles of PhDs, postdocs, or academic assistants. Nearly all journals heavily rely on volunteer work with only very few journals being able to financially compensate editors, editorial managers, assistants and other contributors. Relying on volunteers also means that most journals do not have the capacities to acquire the specialised knowledge needed in some areas of open access publishing such as IT and legal aspects. Among the services outsourced, the most common are design, hosting, and IT development ( Hahn et al. 2023, 8).

The authors also explored the motivations of the editors with recourse to qualitative data analysis of interviews with 22 journals. They found that editors are motivated variously by OA, by growing the impact and reach of their journal and by the ‘enhanced autonomy’ of running their own journals and having control of the content. Nevertheless, their main challenges were relating to funding (or a lack thereof ), overreliance on volunteer work, and the continual need to balance autonomy with standardisation ( Hahn et al. 2023, 18).

Community-led forms of OA are also important from a student-led publishing perspective. Many institutional journals are run by students across undergraduate and postgraduate levels. For example, Uigín and colleagues detail the establishment of a student-led journal in Irish language studies that forms part of the MA programme at the National University of Ireland in Galway. They describe the pedagogical benefits of engaging with the publishing lifecycle and ‘the mechanics of academic writing – editing, copyediting, formatting’ ( Uigín, Higgins, and McHale 2015). Similarly, Wojturska explains that student journals are ‘set up to highlight the work of students but are also spaces for students to engage in and contribute to their academic field in a concrete and professional way’ ( Wojturska 2022). They are also useful for grounding students in academic standards and ethical conduct in research dissemination ( Buckland 2015). Student journals are beneficial, then, for both their academic contributions and their ability to give students experience of a range of technical and organisational skills.

In building on this literature, we are adding an institutional dimension that seeks to offer both a picture of the kinds of community-led publications that exist at the University of Cambridge and a deeper understanding of their motivations. We are aware that Cambridge is a comparatively well-resourced, research-intensive institution in a Global Northern/anglophone context. We are keen not to generalise out from the experiences presented here but instead seek to learn why and how these journals come about and how a wider network of community-led publishers might be supported. In building this argument, we hope therefore to make a case for supporting the growing ecosystem of CPPs and associated publishing projects worldwide.

## 2. Methods

### 2.1 Sample

Through an initial landscape analysis, we mapped the varied community-led publishing projects across the University of Cambridge that we could find and sorted them according to high-level categories (e.g. date established, discipline, publication frequency, editorial leadership, OA model). A total of 34 CPPs were identified. We then qualitatively explored the practices and motivations of these CPPs within Cambridge by purposefully selecting projects from a range of disciplines and inviting editorial members to be interviewed. Following this review, members of the editorial teams (e.g. founders, editors-in-chief, managing editors) of 20 journals were invited via email to participate in an interview. A final sample of 10 participants from seven different journals were interviewed.

### 2.2 Procedure

All interviews were semi-structured and conducted in English between September 2023 and February 2024 by the second researcher. They took place on Microsoft Teams at a time convenient to participants and were guided by a set of pre-specified questions (all research material and data can be found at Moore and Wigdorowitz 2024). While interviews started with general questions about the CPP and participants’ involvement in it, aspects about openness, motivation and support, the order, phrasing and types of questions varied in response to how the conversation unfolded. Follow-up and novel questions arose in some conversations as necessary. Interviews lasted between 32 to 58 minutes (mean = 45.21 minutes) and were audio-visually recorded and transcribed.

We outsourced the services of a transcription company to transcribe the interviews. Following receipt of the transcripts, we thoroughly checked them against the original recordings to ensure we had an accurate record of the discussion. During the initial check, where names or other identifiable information was mentioned, these were highlighted and flagged in the document in accordance with UK Data Service’s guidance on anonymising qualitative data. ^1^ Participants were sent a copy of their interview transcript for approval prior to any analysis or publication. They were asked to clarify, using track changes or highlighting, any content and/or anonymise the transcript further as needed. We informed them that where any identifiable information was mentioned, it would be redacted from the published version and that the final transcript would not include their name in the file title or elsewhere. Interview questions and transcripts (where consent to share was provided) are publicly available in the Cambridge institutional repository, Apollo, ^2^ although video recordings will not be shared beyond the project team.

### 2.3 Data analysis

Interview transcripts were analysed through inductive content analysis, in which emergent themes are derived from data rather than being predetermined ( Elo and Kyngäs 2008). Such a methodological approach was taken because of the limited research in this area and because we wanted the data to drive the results. The interviews were systematically reviewed by each researcher numerous times to identify common themes. In cases of ambiguity or disagreement, the researchers discussed these in detail until consensus was reached. Furthermore, this research is presented according to the Standards for Reporting Qualitative Research (SRQR) checklist ( O’Brien et al. 2014).

### 2.4 Ethics and consent

Before conducting this research, ethical clearance was obtained from the Cambridge Higher Education Research Ethics Committee on 25 May 2023 (REF: 2023.LT.52). The project began on 1 August 2023. Participants who wished to take part in the interview portion of this study received an information sheet and signed a written consent form prior to participating ( Moore and Wigdorowitz 2024). Written and signed informed consent was obtained from all participants. We only shared anonymised transcripts on Apollo where we received permission to do so (and we failed to obtain final permission to share the full transcript of interview 3 although consent was obtained to quote portions of the interview). The study adheres to the Declaration of Helsinki.

## 3. Results and Discussion

### 3.1 Landscape analysis

Thirty-four CPPs were identified across a range of disciplines (for an overview see Table 1, Moore and Wigdorowitz 2024). As mentioned, this list is likely not exhaustive but represents a significant proportion of community-led publishing initiatives in Cambridge. Information about each CPP was taken directly from what was available on their websites. It may be likely that some information is inaccurate, outdated, missing or has since changed, but it nonetheless provides a snapshot of these journals at Cambridge.

Our analysis shows that the first CPP was established in 1840 ( *Proceedings of the Cambridge Antiquarian Society*) and the most recent CPP was established 184 years later, in 2024 ( *Cambridge Journal of Climate Research*), suggesting that this publishing tradition is longstanding in Cambridge. The disciplinary coverage is varied and diverse, with a large contingent of CPPs covering Arts, Humanities and Social Science disciplines and some publishing as magazines or encyclopedia entries. The majority of initiatives classify themselves as journals, but we use the term community-led publishing project (CPP) to broadly capture all initiatives. Some CPPs are affiliated with societies or departments, while others appear to be standalone initiatives. Most are OA ( *n* = 30, 88.2%) and 5 (14.7%) are listed on the Directory of Open Access Journals (DOAJ).

### 3.2 Qualitative analysis

A total of nine semi-structured interviews (with 10 participants) were conducted online with representation from seven diverse journals across Cambridge. Table 2 provides an overview of the interviews, including information about the CPP, number of respondents per interview and length of interview. Note that two interviews were conducted each for the *Cambridge Journal of Human Behaviour* and *Journal of Trainee Teacher Educational Research* – one with the founder and the other with the then editor-in-chief. In addition, one interview comprised two respondents ( *Cambridge Journal of Visual Culture*) while all the others had one respondent. All respondents were given the opportunity to review their transcripts prior to analysis and publication, and five were amended in some way. The approved transcripts are shared on Apollo ( Moore and Wigdorowitz 2024).

**Table 2.** Overview of the interviews.

Interview	Community-led publishing project	Number of respondents	Length of interview	Edits made to transcript
Interview 1	Archaeological Review from Cambridge	1	0:49:52	No
Interview 2	Cambridge Journal of Human Behaviour (1)	1	0:58:08	No
Interview 3	Languages, Society and Policy	1	0:46:42	No *
Interview 4	Cambridge Journal of Law, Politics, and Art	1	0:39:57	No
Interview 5	Proceedings of the Cambridge Antiquarian Society	1	0:42:50	Yes
Interview 6	Journal of Trainee Teacher Educational Research (1)	1	0:32:59	Yes
Interview 7	Cambridge Journal of Human Behaviour (2)	1	0:46:55	Yes
Interview 8	Journal of Trainee Teacher Educational Research (2)	1	0:45:45	Yes
Interview 9	Cambridge Journal of Visual Culture	2	0:46:17	Yes

*Note.* Numbers in parentheses indicate the order of the interview for CPPs where more than one interview was conducted.

* We did not receive permission to share the full transcript, only selected quotations.

The findings are presented across five main themes. These include (1) the *structure* of CPPs, including ‘what’ they encompass, (2) the *motivations* for ‘why’ they exist and ‘why’ people get involved with them, (3) ‘where’ the published content is available and accessible in terms of its *openness and closure*, (4) issues surrounding the *aspects of publishing* and the processes involved in ‘how’ this is done, and (5) the types of *support* needed by ‘who’ to continue and sustain the CPPs over time. A summary of the themes and sub-themes are presented in Table 3. Themes are not completely mutually exclusive and can overlap with other themes, but we have attempted to organise them in a systematic and meaningful way to capture the discussions that arose from the interviews. Each theme and corresponding sub-themes will be discussed in turn. We have identified illustrative quotes for each theme and have sought to contextualise these quotes where possible.

**Table 3.** Summary of findings across themes and sub-themes.

Theme	Sub-theme
Structure - ‘what’	Responsibility
	Voluntary nature
	Scope
Motivation - ‘why’	Dedication and investment
	Uniqueness and value
	Care for authors and communities
	Pedagogical and professional development
Openness and closure - ‘where’	Open access
	Persistence of print
Aspects of publishing - ‘how’	Care for the craft of publishing
	‘DIY’ approach
	Professionalism and publishing standards
Support - ‘who’	Community outreach and exposure
	Technical and financial support from the university
	Handover and preservation

**3.2.1 Structure**

The following theme describes respondents’ responses to how CPPs are structured, particularly who is responsible for them, what forms of labour support these initiatives and the various scopes of projects.

***3.2.1.1 Responsibility***

Our results suggest that CPPs in Cambridge are predominantly run by students, but some are also run by current or retired academics:

‘everything is just run by students’ (int. 1)

‘It’s a student-led journal’ (int. 9)

‘I could never have done this before I retired, I would simply not have had the time or I certainly wouldn’t have done it so thoroughly’ (int. 5)

This is unsurprising given the DIY nature of the publishing initiatives we identified, which have no involvement from professional publishers. However, it would be worth contrasting the labour undertaken for these kinds of initiatives with those supported by professional publishers. Much of academic publishing, not just editorial but many production tasks including copyediting and formatting, are now undertaken by unpaid academics and so it is important to not assume that professional publishing means significantly less work for academics ( Vuong 2022).

***3.2.1.2 Voluntary nature***

CPPs rely on the voluntary labour and efforts of their communities who run them, which is akin to many traditional editorial contributions across the academic publishing sphere ( Wigdorowitz et al. 2024):

‘It’s completely voluntary’ (int. 1)

‘Reviewers don’t get paid, this is all voluntary work as well’ (int 2)

‘completely composed of voluntary members, and mainly students’ (int. 7)

‘It’s completely voluntary. We just don’t have access to enough funding to be able to compensate people, essentially’ (int. 9)

‘we just ran it on a shoestring.’ (int. 3)

There is also space for a more formal workload model which includes engagement with the journal as part of one’s work allocation:

‘part of our workload allocation accounts for that work. So for me, for [administrator] and for all the editors, the compensation is in terms of allocation of workload’ (int. 8)

However, the primary reason for the existence of these CPPs is the vested interest and free labour of individuals who choose, through no obligation, to give of their time to run them. It appears that university departments do not provide specific in-kind support for editors to manage CPPs, nor do they provide financial support for infrastructure or associated costs.

***3.2.1.3 Scope***

CPPs have a wide-reaching scope with various inclusion/exclusion criteria of who gets to publish in them and who can hold editorial positions. Some CPPs have a Cambridge-based focus, publishing only works by students or researchers at the University, while others have expanded to include content from contributors outside of Cambridge. The scope of CPPs appears to be in flux and constantly evolving, with no stringent and unwavering criteria as the needs and purpose of them change. This is evident both in terms of CPPs’ editorship as well as the content and authorship:

‘very much began as a sort of Cambridge student initiative, and since then it has grown. There are a number of other people, myself included, working on it, who were at Cambridge and have now graduated, aren’t studying there anymore or working or things like that.’ (int. 4)

‘the first issue were all Cambridge except for one, maybe. Second issue were half Cambridge. By the third issue, we had one Cambridge, and then … sorry, the second one were just LSE [the London School of Economics and Political Science], I think, UCL [University College London], and then, when we started going international, we had one from Bangladesh, Dhaka, we had one from New York, we had one from Utrecht in the Netherlands.’ (int. 2)

The transitory nature of community-led publishing also made it challenging to identify and categorise CPPs. Editorship often changes between institution and so CPPs are not always based at Cambridge in perpetuity. This is another reason why the CPPs identified should not be considered exhaustive or complete for an extended period of time. The list is a simple snapshot of an ever-changing ecosystem that stretches far beyond the University of Cambridge itself.

**3.2.2 Motivation**

There are numerous reasons as to why people get involved in CPPs, but it is clear that there is a strong sense of care that people have for these initiatives and for the communities they serve.

***3.2.2.1 Dedication and investment***

All respondents had a strong sense of dedication and invested a great deal of time, effort and sometimes personal finances into their CPP. They found them to be labours of love, a common theme throughout scholar-led approaches to publishing ( Pia et al. 2020):

‘I think I was always working on the journal, every day, for a year, and I didn’t take a single break, because there were constantly things coming in, either from the Cambridge Libraries, or a new submission, or something to deal with, a discipline, or just getting things done, figuring things out, learning about journals, going to these events, trying to talk to people, see who else was interested in the journal.’ (int. 2)

‘it’s very much all hands-on-deck kind of structure. So anything you can, you sort of can be doing you are doing. So there’s definitely a lot of changes, a lot of sort of problem solving on the fly, different people doing different things.’ (int. 4)

‘it took time and energy to do that could have been given to other things. But I mean, I thought it was a worthwhile idea so, you know, I thought it was worth putting some time into getting it going.’ (int. 6)

While respondents clearly demonstrate a keen desire to give their time and resources to CPPs, an important distinction should be made about the potentially exploitative nature intrinsic to this type of publishing initiative, which can also be compared to the diamond OA movement in general. Positioning publishing as a labour of love can emphasise that it is work taken not as part of one’s job but more as a hobby or extracurricular activity. The work for CPPs can be recognised and institutionally supported, as some presses show, or it can be something unrecognised and undertaken for its own sake irrespective of remuneration or recognition. While this is not necessarily different to publishing work more generally, the work for CPPs is potentially more expansive than that within traditional publishing settings because editors are in charge of all aspects of the publishing process, not just the editorial side. This makes it all the more important for institutional supporters of community publishing to understand what this kind of publishing entails and to provide adequate support for it (as discussed below).

***3.2.2.2 Uniqueness and value***

Given the limited alternatives to traditional publishing avenues for academic and academic-related work, CPPs provide diverse opportunities for scholarly communication that overlaps with traditional journals but are also unique in terms of the content (e.g. themed issues), authors (e.g. undergraduates, artists) and/or readership (e.g. policymakers). This uniqueness is where the CPPs’ value lies and is a big reason why people are motivated to be involved with them. They prioritise the communities who may not otherwise have a place to contribute and access specific content:

‘we really want it to be something that the man on the street really, the kind of person who would read The Economist or some kind of magazine like that, would be picking up you know for sort of general interest and consuming it just as a kind of media object. Certainly students as well, early career academics. But we conceive it as broadly as possible.’ (int. 4)

‘In terms of what we publish in the journal, though, it tends to be academics, or curators, or artists, although we do also publish some student writing… Currently, we’re on the third issue. Each issue has a theme, around which everything that’s in there centres. Its focus includes essays, articles, artworks, and interviews.’ (int. 9)

‘because a lot of undergraduates don’t have the opportunity to publish. I mean, unless you get a fantastic score at the end of your year. There’s no real forum, and actually, that was the motivation for me founding it.’ (int. 2)

‘what’s unique about our journal, which we haven’t seen in another journal, is we have interdisciplinary commentaries.’ (int. 2)

‘we also get papers from non-academics or retired people.’ (int. 5)

‘we don’t engage with the primary research. The purpose of the journal is to take research that has already been published and assessed from the experts to see how we can get this then to the public domain and bring out the policy implications. So it’s different.’ (int. 3)

CPPs publish alternative content in addition to traditional journal articles and in doing so, attract diverse contributors and audiences. These features lie at the core of CPPs unique contribution and value. Emanating from their disciplinary communities allows CPPs to publish the kinds of content they feel needs to be recognised or for which there are no other outlets.

***3.2.2.3 Care for authors and communities***

A large motivator for these CPPs was a genuine care for the communities that are being served by their contributions and accordingly being able to give something back to authors and readers in return:

‘I really wanted to create an environment in which people could become, could feel like scientists, I guess. Could feel like they were actually conducting real research, that their work could be published, it could be accessible, and it could be referenced one day.’ (int. 2)

‘when somebody does research, you know, you might do the research only for your own benefit, for your own classroom, your own teaching but if it has repercussions that are more general, there is a kind of obligation, particularly when you’ve inconvenienced people by taking their time and whatever, there is a kind of obligation to kind of give back to the community when you learn something from the process.’ (int. 6)

The CPPs themselves are also considered objects of care. Respondents have high levels of respect for them, regarding them in high esteem and as valuable resources because they have worked conscientiously to ensure this is so. They may have also received positive feedback or high levels of engagement with the CPPs, solidifying their importance:

‘it’s quite renowned in archaeology in general as a peer reviewed journal in archaeology. It’s like any other journal, but it just happens to be run by students, which is, I think, a great opportunity’ (int. 1)

‘I think it really looks professional as well’ (int. 2)

‘people are very positive… really impressed with the appearance, the look, but also who is writing for it.’ (int. 4)

‘It’s been a very strong force for the past 150 years in developing and maintaining history’ (int. 5)

‘one student who reached out to us regarding guidance on how to establish a journal, another journal, the collaboration with the SCAJ journal. And they’ve, I think, viewed us quite favourably.’ (int. 7)

‘there were papers that have reached over 100,000 downloads or hits… there was one paper that was picked up, it got mentioned in an article in the National Geographic.’ (int. 3)

Care sets these CPPs apart from so called ‘cookie-cutter’ journals because they do not have any commercial incentives but are rather nurtured because they are worthwhile in their own right.

***3.2.2.3 Pedagogical and professional development***

There are educational and professional benefits involved in contributing to CPPs in the form of learning about the publishing landscape, practices and industry. Respondents acknowledge the expertise it takes to run and publish CPPs, and because of this, they gain specific skills that are viewed favourably:

‘usually a way for students to get into editing’ (int. 1)

‘it was an opportunity for undergrads to also have the opportunity to review, and edit, copy edit, get involved in the media side, or running talks, which we did for one term. And yeah, or just you know what it means to go through a publishing stage, receive a revise and resubmit, to negotiate with an associate editor’ (int. 2)

‘the idea for the journal is not just to give visibility to the work they do, but also to position the work that PGCEs, initial teacher education programmes do, as part of educational research.’ (int. 8)

The knowledge and experience gained from being involved in CPPs are professionally recognised and can be translated into tangible benefits that extend beyond the duration of involvement with the CPP. In this sense, CPPs provide pedagogical incentives. Being involved in CPPs can also add to one’s professional profile with various skills being gained in publishing and in relation to teamwork, collaboration and problem-solving to name a few:

‘It’s also quite good for the CV. I have to say a lot of people do it for that.’ (int. 1)

‘a good experience in a careers sense, in terms of managing teams. We have over 30 people on the team, so there’s a lot of work that goes into managing that and keeping that running smoothly, and just in dealing with sort of the problems that come up across all aspects. So definitely the active resilience and problem solving I think is quite a benefit in that sense as well.’ (int. 4)

‘Later on, I got in a couple of graduate students to help with that process, telling them that this would be good for their professional development’ (int. 6)

‘a good opportunity to learn about the academic publishing industry, which could be helpful in the future.’ (int. 7)

Overall, there are various and wide-reaching reasons as to why people are involved in CPPs and together these motivations of pedagogy, disciplinary commitment, uniqueness of project and care for community provide a holistic picture of their existence.

**3.2.3 Openness and closure**

The second major theme is about openness (and closure) concerning ‘where’ the published content is available and accessible. Respondents had a complicated relationship with ideas of openness and OA publishing. While many CPPs were described as driven by open and public access to knowledge, these reasons were not always of primary concern. Instead, there is an interplay between openness and closure, reflective of the pragmatic decisions made by CPPs to, on the one hand, prioritise open and experimental approaches to publishing and, on the other hand, to ensure their work is sustainable and long-lasting in the form of a physical object.

***3.2.3.1 Open access***

While OA was not always an explicit motivation of the location and accessibility of the content, the idea of free and public access to knowledge was a noteworthy endeavour and was identified as a necessary means to reach both academic and non-academic readership:

‘I think putting paywalls just limits us. I mean, maybe it might encourage people to submit better manuscripts and… but ultimately, we just care about publishing work. We don’t care about earning … well, at least I don’t, and from what I can tell, [editor] doesn’t, either. We don’t care about earning money and that, yeah’ (int. 2)

‘I think open access is a wonderful idea from the point of view that a lot of the research is publicly funded. And if it’s publicly funded, then it should be publicly available’ (int. 6)

‘it has to be open access because teachers in schools don’t have access to academic libraries necessarily. It’s very rare that they would be able to access an article, an academic journal that is not open access.’ (int. 8)

However, some respondents were cautious and critical of the OA transformation in academic publishing, largely due to its financial feasibility:

‘I think open access is a wonderful idea from the point of view that a lot of the research is publicly funded. And if it’s publicly funded, then it should be publicly available… However, the other side of that is who actually pays for this?… if you have all the material available and you’re not asking authors to pay, I think that’s fine. I think there is a problem in moving to a model where you’re asking authors to pay $200, $500, $2,000 to have their work published in that that’s fine if you’re talking about somebody who’s got a grant and as part of that grant there’s money for publication or if they’re in a university like Cambridge, where there’s an agreement with some of the publishers, at least, that this will be sorted out of general funds. But it does mean that somebody who is a teacher in a school, if they do some useful research, they can’t send it to an open access journal unless they stump up a considerable amount of money. So, I think there is a negative about open access’ (int. 6)

‘in general, academic publishing should be open access and, essentially, open for all, as it makes it more equitable, both to publish and gain access to that knowledge. But it’s especially important when it comes to undergraduates because these students are often not getting large sums of funding, if they’re getting any funding at all. So, if you do have article-processing charges, it’s going to essentially make it infeasible for the vast majority of undergraduates to publish.’ (int. 7)

It is important to note that few of the CPPs described their work in the language of the OA movement, such as through specific definitions of OA, adherence to Creative Commons licensing, the use of OA routes like ‘green’, ‘gold’ and ‘diamond’ and so on. CPPs from our study do not appear to emerge out of the OA movement per se, despite often showing an affinity for public access to knowledge, nor do they necessarily show deep understanding of the political economy of publishing that motivates OA. Rather, OA was (or was not) taken as a standard practice within the current structure of the CPPs:

‘Now that we’re doing sort of rolling online publication it will certainly be much less, you know, it will be much easier to put people’s articles out immediately when they’re finished online. As previously we would only sort of publish online once everything else was finalised.’ (int. 4)

‘there is no alternative way for this because there is no… you know the whole point of this is to make the linguistic research accessible.’ (int. 3)

‘the most recent three volumes are embargoed, which means that they’re not accessible to read to the wider public and that gives the membership some kind of continued limited access. I don’t know how long that system will continue and in recent years it’s run us into problems with open access. There are some ways around this, but in fact we don’t get so many of the open access papers because our journal, being a small, local, British journal, doesn’t get in the A-list of journals for aspiring academics to publish in. So, we’re not the first choice of a young academic trying to make their career.’ (int. 5)

‘So the journal is hosted on our faculty website, so in many ways it’s as if this were like any other thing that you had in a faculty website to showcase pieces of work done by the faculty members right. So, it’s not like we need to pay for a system to help us, so we don’t need to go and pay someone to do it, which means that we don’t need to put paywalls or anything. So it is very straightforward, in terms of costs for it being open access etc. So there was no specific financial reason for making this not open access.’ (int. 8)

‘I think with making it available online, if there was… like that would make peer reviewing more possible… by making it open access, it could be referenced, it could be peer reviewed for further research, and that’s really one of the main reasons that we do want to put it online.’ (int. 9)

***3.2.3.2 Persistence of print***

Along with the practices of online OA, some CPPs also engaged in more traditional practices of print publishing. The printed object is spoken about very highly and connects back to the care for the publishing process identified above. Many respondents spoke of print as ‘beautiful’ and associated it with a sense of tradition, legitimacy or professionalisation, an aspiration or something that was an expectation of their readership:

‘I mean we love the idea of having them openly available. I don’t think we would ever take that away because we don’t want this to be an exclusive journal, especially as it wasn’t born with that intention. It was mostly so that students could put their hands on editing. And so, if students then don’t have access to openly available articles, then we wouldn’t be happy with that. Unfortunately, we do have to have the embargo period because otherwise we wouldn’t be able to print, but there have been talks about having it online only. The older people in the department are very unhappy about that, so we keep printing’ (int. 1)

‘We don’t print, although that is something that I’ve always wanted to do.’ (int. 2)

‘I think the printing is very important, yeah. The idea was to make a beautiful object, you know. And the design is, a lot of thought went into the design to really get something that looks very crisp, you know, very nice to look at, to have high quality reproductions of images. And I think that was an appeal, that was a massive appeal for the contributors as well. Certainly the first two issues for the students, knowing okay, this will actually be printed in a genuine object, rather than just being posted somewhere online where not many people might see it, I think is a huge motivation and something very special for the people who are submitting. So that’s definitely really important to the identity of the journal I would say’ (int. 4)

‘Every subscribing member gets a printed version and many of the members would not read online.’ (int. 5)

‘I thought it was quite special, because it’s something that can be archived, the journal, in its physical format: many these days are online only.’ (int. 9)

The persistence of print and the desire for OA also reveals a tension between openness and the need for certain kinds of closure. Respondents described their reliance on print subscriptions and the consequent need to embargo digital copies as reasons to encourage readers to purchase print copies:

‘they are all open access, but we do have an embargo period of six months because that’s how we sell subscriptions.’ So, in order for us to print and send out volumes we mostly like to print our issues and organise the launch in order that we have subscriptions that get paid to us. So, that basically we are self-sufficient and the university doesn’t give us any funding or anything. But for that to work, we do have a six months embargo period so that the subscribers will be the first one to receive the journal and for six months they will be the only one having it. And then after six months, it’s open. The articles will be online, open access’ (int. 1)

‘All of the articles from our first two issues will remain free, open access in perpetuity. But as I say, for the coming release I think we’re looking to have some kind of individual subscription model or institutional subscription model whereby if there are 100, 80, 100 articles in the volume, maybe 30 of those are open access and then the other 50, there’s a small sort of monthly subscription fee.’ (int. 4)

‘the most recent three volumes are embargoed, which means that they’re not accessible to read to the wider public and that gives the membership some kind of continued limited access.’ (int. 5)

The implementation of OA in these CPPs is often shaped by practical considerations rather than pure alignment with OA movements. While many CPPs embrace OA as a natural or pragmatic practice to reduce barriers to access, they face challenges related to financial sustainability, through print sales and subscriptions, and structural constraints. In addition, print practices persisted in some CPPs because they are a tangible resource and have professional appeal. This highlights the choices made for CPPs regarding the ideal of equitable knowledge dissemination and the practical realities of publishing in a resource-limited environment.

**3.2.4 Aspects of publishing**

This theme illustrates the different aspects of publishing undertaken within CPP approaches, including the processes involved in ‘how’ this is done.

***3.2.4.1 Care for the craft of publishing***

All respondents showed a real care for their publishing contributions, not only in the sense of professional design, but also for the scholarly aspects of their publishing projects, including in copyediting, subject knowledge and processes:

‘I can guarantee that 99% of all references are perfectly written in APA 7, and that’s also another thing I wanted to ensure.’ (int. 2)

‘I didn’t want to publish bad work, let’s say, or poorly-reviewed work. So, we’ve always been very strict with that. If any errors occur, we’re always straight on it.’ (int. 2)

‘we have very good procedures and protocols in place… I think it’s running very organically almost, and it’s very automatic how we do it. And I think the running of it is going well.’ (int. 8)

Such care contributes to the rigour and trustworthiness of the content being published.

***3.2.4.2 ‘DIY’ approach***

CPPs tend to employ a ‘DIY’ approach to getting things done within the confines of their available people capacity and resources. They are reasonably flexibly in how and when content is published and follow different timeframes that work for them and their audiences:

‘publish biannually’ (int. 1)

‘we usually publish October, January, April, July, maybe August’ (int. 2)

‘It’s a sort of irregular timeline really. I mean, the first one came out in summer 2021, and then the second issue was towards the end of, online release was towards the end of 2022, and then the print release was February 2023. And then we’re looking to put out two volumes this year, one around March and the other one around October. But it’s not a, there’s not really a strict regular timeline, it all sort of depends on how many contributors we’ve been in touch with, how large we want it to be.’ (int. 4)

‘it’s kind of an annual thing but on a kind of rolling basis.’ (int. 6)

Some are also limited by the software and journal management services they use, which are typically not made-for-purpose. Yet, even with these restrictions, they make it work and are able to publish high-quality and diverse content, albeit with the content not always reviewed by all CPPs in the strictest sense of peer review:

‘we have a template on InDesign that we tend to use. But really, it’s up to the theme editors what they want to do.’ (int. 1)

‘we do everything on Google Drive, which is supposed to be well encrypted and quite secure.’ (int. 2)

‘I designed a template but even so, not everybody used it and not everybody used it perfectly.’ (int. 6)

‘currently, we are just storing them on Google Drive. As part of our partnership with the Lyrasis, we’re hoping to use a large amount of the funding to get a manuscript management software.’ (int. 7)

‘the journal is hosted on our faculty website’ (int. 8)

‘it’s very straightforward because we have a very, very specific template from the start. So the trainees have to submit in a particular template. It’s one of those.doc file templates that they can just copy and paste the stuff and work in the template to avoid nightmares.’ (int. 8)

‘I think there’s a lot of flexibility, really, with what people write and what we publish. Yeah, it’s sort of a split between interviews between the editors and the contributors that they’ve chosen, or just essay or articles that the contributors have written themselves. But, other than that, it’s quite flexible. We’ve, sort of, got a rough word count of 1,000 to 2,500 words, but yeah, the format, I think, is quite open.’ (int. 9)

‘in some cases I’ll send it out to referees. If it’s on a topic that I’m not particularly competent to assess myself, if it’s a really quite substantial paper.’ (int. 5)

‘although I understood the logic of saying well, let’s make it peer reviewed, I thought that was a terribly unnecessary complication both in terms of requirements on the students and also on the administration because if you have a peer review system, that immediately adds a whole load of complexity to what you’re doing. So, I mean what came out of it was a kind of compromise. I designed a peer review form and said to the peer reviewers, “This work has already been assessed and judged to be a good standard so we’re not asking you to do an evaluation, basically, on whether it’s any good. But would you look for things that might need changing?” such as the person should have anonymised the report so that no individual child they spoke to could be identified. They should have all the referencing properly in place, you know, things like that.’ (int. 6)

The ‘DIY’ approach of these CPPs illustrates how adaptive and resourceful they are even with limited capacities. They often operate on flexible publication schedules tailored to editorial, contributor and/or audience needs. Despite software constraints, CPPs manage to produce high-quality and diverse content. Editorial practices vary, with some journals employing formal peer review or simplified processes focused on quality assurance rather than rigorous academic evaluation. Flexibility also extends to content format and length, allowing for varying solutions and levels of freedom among contributors.

***3.2.4.3 Professionalism and publishing standards***

Although many CPPs do what they can to publish content with the limited resources they have, many try to adhere to high publishing standards and professionalism comparable to that of traditional publishing models, such as having formal peer review and persistent identifiers:

‘it is considered quite well, mostly because it’s peer reviewed’ (int. 1)

‘here, we really take seriously the aesthetics as well, because we are professional in our approach, in our rules, in our ethics, in our guidelines. And we take seriously our reviewing process, and so we also want to make sure that when authors submit and they get to get published, that their work looks professional, and that it’s recognised, and the journal is established, with ISSN, and hopefully DOIs soon.’ (int. 2)

‘the team thought if we were going to do a journal, there should be a sense of formal peer review.’ (int. 6)

‘for the policy papers, we get a DOI so they’re also uploaded on Apollo and that’s where we get the DOI from. So, from that point of view, people actually have submitted them for their REF impact publications, sorry, submissions.’ (int. 3)

‘for two years now we have DOIs for the articles… they are deposited on Apollo and now we have DOIs for the articles so we can get a little bit of traction on that side of citations etc’ (int. 8)

The varying approaches CPPs bring to publishing illustrate the complexity and heterogeneity of this ecosystem. We should therefore avoid one-size-fits-all conceptualisations of these publishing models in comparison to traditional journals but rather view them as unique entities with their own methods and structures that sits within a wider publishing niche.

**3.2.5 Support**

It is the case that all publishing outlets require a diverse array of support to run and be sustainable. Support can take different forms but mainly includes financial, technological and human capital ( Brun, Pontille, and Torny 2024). This is particularly salient for publishers who are independent, new and publish content from underrepresented researchers and disciplines. CPPs are no different in this regard and require varying forms of support which largely come from the available resources within their contexts and networks.

***3.2.5.1 Community outreach and exposure***

Support is needed in the form of community outreach initiatives which are key to spreading the word about the CPPs within a broader network. Such exposure allows these projects to share issues, calls for papers and other relevant news as well as to benefit by connecting with others who are involved in similar publishing initiatives:

‘the thing that will be more useful is getting a broader network. So, to get the call for papers out and have more publicity and collaborate more I guess. So, it’s sort of to reach a bigger network. Also, with advertising.’ (int. 1)

‘I think a fair amount of it is from word of mouth, and then we also advertise on social media. And then, in terms of submissions, we also have an outreach team, outreach officers for the various divisions. And then they will directly contact departments, just with a brief blurb, asking a representative from the department if they could just put in their weekly newsletter, an announcement that we’re open for submissions.’ (int. 7)

‘to establish a directory of student-run journals for the university, just so that you can know what the different student-run journals are, whether … if you want to publish with them, or for the various editorial boards of those journals to communicate with each other, as they may be facing similar problems or have opportunities that are relevant to each other.’ (int. 7)

‘just expanding its outreach, and the awareness of the journal, for people to publish with it.’ (int. 7)

‘things like posters, and getting through other departments by reaching out to our secretary to reach out to others would be useful for more people across different subjects to come. And all of us on the team inviting friends from our colleges and elsewhere should help also.’ (int. 9)

Editorial members are also eager to promote and endorse the CPPs that they have worked on. Promotion is realised in a variety of ways, including through launches and events throughout Cambridge to showcase the content, make people aware of the CPP and connect with the broader community:

‘I was talking yesterday to someone from the Intellectual Forum at Jesus College, which was you know a good, productive initial discussion about the possibility of potentially hosting, co-hosting a launch event for our October volume at the Intellectual Forum.’ (int. 4)

‘We have two conferences each year, Spring and Autumn’ (int. 5)

‘I hope by June time we should be having our launch party, when we have the physical journal in print.’ (int. 9)

Effective community outreach and promotion are crucial for the growth and sustainability of CPPs. Building broader networks through various means as well as through collaborations helps raise awareness, attract submissions and engagement with content, and fosters the opportunity for connection with similar initiatives throughout the university. Respondents emphasised the importance of events, launches and creative strategies to showcase their work and engage both contributors and audiences. Establishing directories and building communication among CPPs can further enhance their collaborative efforts and provide a platform to address shared challenges, ultimately strengthening the community and increasing visibility.

***3.2.5.2 Technical and financial support from the university***

Quite possibly the most significant form of support for these CPPs is having the financial means to continue but not profit off the output. Funding sources noted by respondents come from university departments, colleges and clubs, subscriptions, sales of issues and external sponsorships:

‘we only got money from subscriptions, selling issues and publisher’s licence.’ (int. 1)

‘There’s money obviously from sales, sales of the journal.’ (int. 4)

‘since the largest problem that I see is funding, if various departments or institutions within the university, or the university itself, could establish some sort of fund for open-access publishing within the university, I think that would be greatly beneficial.’ (int. 7)

‘it’s not a profit-driven journal at all, but we probably do want to hopefully see if we can make the printing process a little cheaper, to make some turnover profits for next year’s team start off with. Though still, funding at the moment will come out of the generosity of colleges and department’s support.’ (int. 9)

In addition, other forms of support that usually require a financial investment but could be covered through the university to offset these costs could come in the form of workload adjustments, infrastructural support to host the journals, and promotional, administrative and educational support from the libraries and academics with publishing experience:

‘if that is something the library could support us with, promotion of the content’ (int. 3)

‘I would have liked to spend an afternoon per week or something along those lines, to… because I think if I could have an afternoon per week, I could promote the papers better, get more content, do things much faster. But I’m afraid it’s becoming an afternoon per month’ (int. 3)

‘we have the IT support as well as part of this infrastructure of the faculty webpage. And then we have the support from the Apollo team at the university with the depositing. But it’s not financial support, we have workload support if that makes sense.’ (int. 8)

‘if there are actually a set of editors that are actually working, like are actually working as editors and will be available to sort of give, have a meeting to give an idea of the editing process or tips, that would be quite useful’ (int. 1)

‘if there could also be advisory committees, or voluntary advisory committees, by various academics throughout the university, whether they’re members of different departments or those who are experienced in publishing who are, maybe, editors or reviewers for various journals within their respective fields, or those who work at the Cambridge University Press and are very knowledgeable about the publishing industry, if there could be just an advisory board where, if journals have any questions about various aspects of the publishing process, or they need someone to contact with different areas of specialties, that they can contact them easily and know where the contact information is and that they’re willing to help and give advice.’ (int. 7)

In addition to financial aspects, then, CPPs highlight the social need to share and receive advice, alongside more technical guidance from experts already housed within the university. Such comprehensive support would significantly enhance the efficiency and impact of CPPs while ensuring their longevity.

***3.2.5.3 Handover and preservation***

CPPs are particularly dynamic in their turnover of editorial members, with tenure ranging quite significantly from CPP to CPP:

‘according to our constitution, the general editor changes every time we publish a new volume’ (int. 1)

‘we usually have a general editor every six months. And the general editors oversees the editing process and the publishing process’ (int. 1)

‘We decided that the editor-in-chief post should stick to one year only. Managing editors should be termly, although they could be re-elected, or maybe every two terms, and then associate editors was termly. And that worked very well. We never really had any issues with that side. Of course, you know undergrads, they drop in, drop out, because it’s in terms of workloads, whatever.’ (int. 2)

‘I’ve been in the role for 10 years, it hasn’t changed particularly. I took over from the previous editor. If you look back at the history you can see there’ve been two or three of us who’ve been long-term editors and there have been, then sometimes interregna, where they haven’t found a new long-term person instantly.’ (int. 5)

‘theoretically, my position can be held for two years, and managing editors can hold their roles for up to three years. So far, no one has held any of those roles for the maximum amount of time, and the time limits were just implemented at the beginning of this year, with amendments to our constitution. And then the reviewer and associate editor positions don’t have any time limits.’ (int. 7)

Given the dynamism of the editorial roles of these CPPs, a smooth handover is crucial to their preservation. This frequent turnover necessitates smooth handovers to maintain continuity, yet challenges can arise, such as finding successors or helping new editors navigate steep learning curves:

‘you get to a point where, especially when I was retiring, you really need somebody else to take it over.’ (int. 6)

‘I think in future it might make sense to have an editor who actually did all the copy editing and typesetting themselves, which some journals do.’ (int. 5)

‘when people take on new roles, they may be a bit unfamiliar with what’s expected of that role and what the duties are, so sometimes that can be a bit of a challenge. Just, I guess, the learning curve for them, which is understandable.’ (int. 7)

‘That team has been consistent for a while now. We have had the same members in that team with a couple of extra ones joining, but with a core team working in the journal for more than five years now. Which means that even when [editor] retired and we had this gap, in terms of editor-in-chief, those people knew the journal very well and they just kept working with that.’ (int. 8)

Despite constant transitions, many CPPs achieve stability through dedicated core teams and plans for future recruitment, allowing for the preservation of institutional knowledge and operations. Overall, their resilience relies on structured transitions and passed-down wisdom.

## 4. Summary and concluding recommendations

Within this article we have described a rich ecosystem of community-led publishing projects at the University of Cambridge. These projects reflect an array of practices and motivations with varying degrees of professionality and technical sophistication. What binds them is the DIY approach – the fact that they run these projects themselves and for the most part stand outside the traditional publishing industry and the standards imposed by professional publishing. This means that community-led publishers are able to foreground their own values and practices as part of their work, not least due to the fact that they are free of the commercial restraints of financial sustainability, which offers a degree of freedom to experiment and also less of an impetus to publish with regularity or consistency.

Arising out of this freedom from commerciality are the practices of care for community and scholarship that exist with CPPs, including how these kinds of approaches to publishing facilitate new kinds of publication, different approaches to authorship and alternative audiences beyond the confines of traditional academia. Through this article, we have tried to emphasise less of a coherent approach to CPPs and more the fact that being ‘community-led’ permits a degree of control that has been lost in publishing over the past thirty years. We hope to make the case for supporting this landscape precisely because it could facilitate experimentation and exploration into what the futures of publishing could be. This is to say that CPPs have intrinsic scholarly value rather than value for any particular outcome or practice. They should therefore be nurtured precisely because they illustrate what happens when publishing is placed under greater scholarly control.

Yet we have only just scratched the surface of this landscape and are quite sure that many more CPPs exist that we were unable to uncover. This is perhaps because these publishers do not tend to define themselves by their institution but rather their disciplinary connection, meaning that they are not locatable by particular keyword searches or other identifiable features. Many of the CPPs we found were from word of mouth or happenstance, in addition to the small number that appeared in databases such as the Directory of Open Access Journals. This leads to our first recommendation – one which is also mentioned by many of the CPPs we interviewed – that libraries should support these publishers to achieve a minimum threshold of technical and preservation standards to aid discoverability, for example by ensuring they have ISSNs and DOIs or other forms of persistent identifiers. While we do not want to be too prescriptive about this recommendation, there exist a variety of standards that CPPs could aim to adopt, such as the Directory of Open Access Journals mentioned above or the Extensible Quality Standard in Institutional Publishing (EQSIP) defined by the DIAMAS project ( Armengou, Redhead, and Rooryck 2023b). These standards are defined with more ethical approaches to publishing in mind than, for example, those of commercial databases like Web of Science or Scopus. Through technical expertise, universities could support CPPs to reach the technical standard they desire, keeping in mind that the decision to standardise should not be onerous on the part of the academics and students who organise each project.

Alongside the standardisation required, universities should also look to build capacity around community-led publishing, particularly in relation to the turn to diamond OA as an alternative model for publishing that does not rely on the services of a private, unaccountable industry for its work. Building this capacity means ensuring that the necessary training documentation, software and advice is in place for university members who want to start their own publishing projects. Many universities now have staff members dedicated to supporting CPPs within the university, which is a positive step for such projects. Universities should also work with CPPs to ensure their processes are documented so that new editors can transition smoothly, but also to provide an audit trail for the publishing work being undertaken. We have seen how editorial boards are constantly changing and so adequate documentation would offer a degree of continuity between the revolving door of editorial leadership.

Similarly, universities should recognise the time and effort that academics and students spend on their publishing projects, ideally as a form of service that contributes to broader intellectual and collegiate life of the university and beyond. This point is made by Adema and Moore in an article where they argue that universities should offer one day a week of ‘unstructured autonomous time’ for the academics who work on publishing projects, which would allow a flourishing ecosystem to grow and would also encourage academics to divert their labour from extractivist commercial publishing houses towards in-house initiatives ( Adema and Moore 2024). Adema and Moore also argue that publishing, particularly in Arts, Humanities and Social Science disciplines, can be an extension of one’s scholarly research activity, not separate from it, making it all the more important to support the labour of those already engaged in this activity. To be clear, we do not want to be prescriptivist about community-led publishing and are not making a case that all publishing should be organised within the university in this way, which is as unfeasible as it is undesirable. Yet we advocate that community-led publishing is valuable and worthy of support as a mode of production with particular ethical benefits to scholarly research and communication.

Finally, we recommend that universities nurture the communities behind these projects by bringing CPPs together to share advice and support each other’s work. Some CPPs described feeling isolated from other journals and having to learn everything about the publishing processes themselves and largely from scratch. While some of these issues can be mitigated through better documentation and software, they also point to a social need that CPPs have to share with one another to feel part of a community. Journal collectives such as the Free Journal Network, the Library Publishing Coalition and the Radical Open Access Collective ^3^ are examples of the kind of network that universities could organise across and between universities to foster mutual reliance and advice sharing between CPPs. Providing such structures is a cost-effective way of supporting the growing ecosystem of CPPs and would also act as a presence to attract more researchers to explore the benefits of DIY forms of publishing.

## Ethics and consent

Before conducting this research, ethical clearance was obtained from the Cambridge Higher Education Research Ethics Committee on 25 May 2023 (REF: 2023.LT.52). The project began on 1 August 2023. Participants who wished to take part in the interview portion of this study received an information sheet and signed a written consent form prior to participating ( Moore and Wigdorowitz 2024). Written and signed informed consent was obtained from all participants. We only shared anonymised transcripts on Apollo where we received permission to do so (and we failed to obtain final permission to share the full transcript of interview 3 although consent was obtained to quote portions of the interview). The study adheres to the Declaration of Helsinki.

## Data Availability

University of Cambridge: Research data supporting: ‘“I just very much love the journal”: Understanding the community-led publishing landscape at the University of Cambridge’. https://doi.org/10.17863/CAM.111985 ( Moore and Wigdorowitz 2025). The project contains the following underlying data:
• Interview 1.pdf (transcript) • Interview 2.pdf (transcript) • Interview 4.pdf (transcript) • Interview 5.pdf (transcript) • Interview 6.pdf (transcript) • Interview 7.pdf (transcript) • Interview 8.pdf (transcript) • Interview 9.pdf (transcript) • Interview Questions.pdf •
participant_consent_form.pdf (106.7 KB) •
participant_information_sheet_final.docx • Interview questions.pdf (92.61 KB) University of Cambridge: Research data supporting: ‘“I just very much love the journal”: Understanding the community-led publishing landscape at the University of Cambridge’. https://doi.org/10.17863/CAM.111985 ( Moore and Wigdorowitz 2025). This project contains the following extended data:
•
Table 1. docx (23.12 KB) Data are available under the terms of the Creative Commons Attribution 4.0 International license (CC-BY 4.0).

## Footnotes

^1^

https://ukdataservice.ac.uk/learning-hub/research-data-management/anonymisation/anonymising-quantitative-data/

^2^
Eight of the nine transcripts are available on Apollo ( Moore and Wigdorowitz 2024).

^3^

https://freejournals.org/, https://librarypublishing.org/, and https://radicaloa.postdigitalcultures.org/,respectively (last accessed 10/01/2025).
