# Supplementary material for: “I just very much love the journal”:  Understanding the community-led publishing landscape at the University of Cambridge
Source: F1000Res. 2025 Jul 23;14:266. Originally published 2025 Mar 5. [Version 2] doi: 10.12688/f1000research.161163.2 (PMC12291055; doi:10.12688/f1000research.161163.2)
Supplement: Peer review history

# Reviewer report: Reviewer response for version 2

**DOI:** 10.5256/f1000research.185245.r400154

**Published:** 2025-09-16

**Authors:** Leigh-Ann Butler, Madelaine Hare

**Affiliations:** 1 University of Ottawa, Ottawa, Ontario, Canada

The authors describe their amendments as clarifying methodological and definitional issues. To address the latter, a table has been added to clarify the differences between CPPs and professional publishers. The inclusion of Table 1 is effective at more clearly stating how they conceptualize CPPs and their associated criteria. They have also pointed to literature on care ethics and labour, alongside the political economy of publishing. As previously noted, the authors might consider providing a separate document with just the interview questions to facilitate reuse. We thank the authors for their study; this is an important and wonderful contribution to the area of community-led publishing.

Is the work clearly and accurately presented and does it cite the current literature?

Partly

If applicable, is the statistical analysis and its interpretation appropriate?

Partly

Are all the source data underlying the results available to ensure full reproducibility?

Yes

Is the study design appropriate and is the work technically sound?

Partly

Are the conclusions drawn adequately supported by the results?

Yes

Are sufficient details of methods and analysis provided to allow replication by others?

Partly

Reviewer Expertise:

open scholarship, scholarly communication, library publishing, academic publishing, science policy, bibliometrics

We confirm that we have read this submission and believe that we have an appropriate level of expertise to confirm that it is of an acceptable scientific standard.

# Reviewer report: Reviewer response for version 2

**DOI:** 10.5256/f1000research.185245.r400151

**Published:** 2025-09-12

**Authors:** Abdelghani Maddi

**Affiliations:** 1 CNRS and Sorbonne University, Paris, France

The authors have thoroughly and constructively addressed all the concerns raised in my initial review. The revised manuscript is now clearer, more rigorous, and better grounded in the relevant literature. I have no further comments or suggestions, and I would like to sincerely congratulate the authors for the quality and care they have put into this manuscript.

Is the work clearly and accurately presented and does it cite the current literature?

Partly

If applicable, is the statistical analysis and its interpretation appropriate?

Yes

Are all the source data underlying the results available to ensure full reproducibility?

Yes

Is the study design appropriate and is the work technically sound?

Yes

Are the conclusions drawn adequately supported by the results?

Yes

Are sufficient details of methods and analysis provided to allow replication by others?

Yes

Reviewer Expertise:

Scholarly Communication, Scientometrics, Open Science, Research Evaluation, Research Integrity.

I confirm that I have read this submission and believe that I have an appropriate level of expertise to confirm that it is of an acceptable scientific standard.

# Reviewer report: Reviewer response for version 2

**DOI:** 10.5256/f1000research.185245.r400152

**Published:** 2025-08-30

**Authors:** Chun-Kai (Karl) Huang

**Affiliations:** 1 Curtin University, Bentley, Western Australia, Australia

No further comment.

Is the work clearly and accurately presented and does it cite the current literature?

Yes

If applicable, is the statistical analysis and its interpretation appropriate?

Yes

Are all the source data underlying the results available to ensure full reproducibility?

Partly

Is the study design appropriate and is the work technically sound?

Yes

Are the conclusions drawn adequately supported by the results?

Yes

Are sufficient details of methods and analysis provided to allow replication by others?

Yes

Reviewer Expertise:

Open Knowledge, Open Science, Open Access, Statistics, Data Science, Bibliometrics, Scientometrics, Probability Theory

I confirm that I have read this submission and believe that I have an appropriate level of expertise to confirm that it is of an acceptable scientific standard.

# Reviewer report: Reviewer response for version 1

**DOI:** 10.5256/f1000research.177159.r375559

**Published:** 2025-05-28

**Authors:** Leigh-Ann Butler, Madelaine Hare

**Affiliations:** 1 University of Ottawa, Ottawa, Ontario, Canada

This exploratory study provides a snapshot of community-led publishing projects (CPPs) at the University of Cambridge. Employing qualitative methods through a landscape analysis and interviews, the authors aim to understand the existing CPPs, their publishing process, and the motivations of the journals. The study identified a total of 34 CPPs at Cambridge, resulting in an interview sample of 10 journal editors. Despite the small sample size, the data is rich, and much can be inferred from participant expertise and experiences. A key strength of the study is how it centres the voices of those directly involved in CPPs.

 This topic is timely and relevant as diamond open access is increasingly seen as playing a key role in an equitable and sustainable scholarly publishing future. The authors emphasize the importance of diamond OA in addressing the economic challenges of the monetization of scholarly publishing, including the rise of APCs and transformative agreements. CPPs play a significant role in this diamond ecosystem, but the voluntary nature of editorial work and the limited resources create a precarious environment. 

 There are several conceptual and methodological ambiguities which make it difficult to determine if the aims of this research have been achieved. With a few clarifications and minor revisions, this study will offer two key contributions: first, by offering methodological pathways for other studies investigating CPPs, especially through the open availability of their dataset, and second, by offering useful recommendations for future research.

 1. Is the work clearly and accurately presented, and does it cite the current literature?

 The authors could more clearly define their conceptualization of CPPs and their associated criteria. The operationalization of their definition in their identification and screening of CPPs is vague; clarification within the study’s context will elucidate what valuable insights are gained. 

 The authors could add a Research Objectives section to clarify their aims. In the introduction it is noted that they aim to understand which CPP exist, how they publish, and their motivations. Following this, they state they are interested in the kinds of practices CPPs engage in, why they do what they do, and how their work might be supported in the university and beyond. A tighter framing would help the reader assess whether the study’s goals were met and how each were addressed.

 Minor adjustments to their Background section could improve clarity:

- The sentence “Community-led publishing has been an important part of OA publishing, and a flourishing ecosystem of community-led publishers exist globally today...” would benefit from an example as community-led publishing varies globally.
- “Diamond OA and community-led publishing are not synonymous...” There are various diamond criteria, such as those outlined by DIAMAS’ Diamond Open Access Standards (DOAS) outline. Citing these could clarify the distinctions between diamond and community-led publishing if they do not always overlap.

This study could also contextualize itself within the academic environment of the UK, particularly within Cambridge. It could be explicitly stated that the authors work within this institution and are familiar with community led publishing projects and environment or elaborated that the UK is a growing space for these projects; for the latter, the authors might consider dedicating a new paragraph before this one. The UK is an interesting case study due to its research evaluation models and its shift away from transformative agreements. The authors note they aim to explore how CPPs can be supported within Cambridge and beyond, and so space dedicated to contextualizing their findings in this regard could make them even more effective, particularly due to the small sample size from one institution.

 2. Is the study design appropriate and does the work have academic merit?

 Although the authors note the study is not and could not be systematic, there are conceptual and practical ambiguities resulting from a lack of specification of the inclusion and exclusion criteria, and definition of CPPs. For example, are CPPs, by definition, open access? This is not made explicit and should be. Many of the characteristics the authors describe (e.g., society journals) can also apply to commercial publishers. Here, the authors have the opportunity to respond to established definitions of CPPs by proposing a new one or revising or expanding current ones through their results by commenting on the (in)compatibilities of their findings with current understandings.

 3. Are sufficient details of methods and analysis provided to allow replication by others?

- How were journals located in the landscape analysis? What was the approach? This could also be a methodological contribution of the paper, so more details would be appreciated.

- A total of 34 journals were identified. What were the inclusion/exclusion criteria? Did both authors agree on these?
- How was the coding conducted between the authors to generate themes?
- In table 2, the role (e.g., editor-in-chief, managing editors) and their status (faculty, student, library workers) could be noted if a criterion of CPPs is that they include various community members.

4. If applicable, is the statistical analysis and its interpretation appropriate?

 In section 3.2.1.1 – when you say “CPPs are predominantly run by students…”, does this mean the journals are student-run (only students perform editorial roles) or that students participate alongside faculty?

 The presentation of results could benefit from streamlining the quotes one after another to improve the readability and flow of the quotes. This section should also include the author’s analysis with a few key quotes, when necessary. 

 5. Are all the source data underlying the results available to ensure full reproducibility?

 Yes, but the authors could provide a separate document with just the interview questions to facilitate reuse (I’m sure other institutions would appreciate adapting them for their own contexts!)

 The second paragraph of the Results and Discussion could be its own section of the paper to better answer the landscape analysis aspect of the study: what are the characteristics of the publications and how do these align with the qualitative findings from the interviews? The authors did do this in one paragraph and focus more on the qualitative findings, but these results could be linked together more effectively.

 In regard to Table 3’s sub-themes, it would be helpful to understand how the authors interpret these themes in relation to CPP (this might be clearer with a more delineated definition), and again, how these themes were generated. For example, “care for the craft of publishing” is ambiguous: what are the indicators used to code?

 Thank you for the opportunity to review this study. We approve with reservations but believe with revisions, the study and its recommendations will be of value and a great contribution to the area of community publishing.

Is the work clearly and accurately presented and does it cite the current literature?

Partly

If applicable, is the statistical analysis and its interpretation appropriate?

Partly

Are all the source data underlying the results available to ensure full reproducibility?

Yes

Is the study design appropriate and is the work technically sound?

Partly

Are the conclusions drawn adequately supported by the results?

Yes

Are sufficient details of methods and analysis provided to allow replication by others?

Partly

Reviewer Expertise:

open scholarship, scholarly communication, library publishing, academic publishing, science policy, bibliometrics

We confirm that we have read this submission and believe that we have an appropriate level of expertise to confirm that it is of an acceptable scientific standard, however we have significant reservations, as outlined above.

# Reviewer report: Reviewer response for version 1

**DOI:** 10.5256/f1000research.177159.r375557

**Published:** 2025-05-07

**Authors:** Mikael Laakso

**Affiliations:** 1 Tampere University, Tampere, Finland

Thank you for the opportunity to review the paper ”“I just very much love the journal”: Understanding the  community-led publishing landscape at the University of  Cambridge”. 

 The study defines and conceptualises community-led publishing projects (CPPs) and conducts a mapping study on the CPPs active at Cambridge University, resulting in 34 CPPs, which is then followed up by qualitative interviews with 10 journal editors connected to the identified CPPs. The interview transcripts are inductively analysed to enable bottom-up identification of themes from the interviews. Over the years there have been many individual case studies written from the perspective of how individual community operated journals are surviving, often with scarce resources, but no study that would draw upon a set of journals that are often loosely coupled with a single institution exists to my knowledge, which in my view is a strong positive for the overall study design.

 The different parts of the paper are sound and well-presented:

 The introduction provides a good introduction into the topic area, which is related to the ongoing research and science policy efforts of diamond open access publishing, institutional publishing, and nonprofit publishing - all of which are relevant to the concept of CPPs that is introduced and defined very well. Resources are up to date and offer glimpses into the different topic areas that relate to this study.

 The methods section is well-prepared and comprehensive, making it possible to fully follow the process that lies behind the paper and the analysis methodology for the interviews. A huge strength of the paper is that most of the interview transcripts are linked to as open data, so that it is possible to read them from start to finish if one would wish to do so, or incorporate them into future research.

 The results presentation is solid and provides a generous amount of quotes extracted from the interviews to support the different identified themes and findings. If anything they could be slightly reduced or shortened to make the paper a bit snappier, some transcript segments are also quoted multiple times for example. Since the full transcripts are available for anyone wanting the complete narratives. In this publication format where print or word counts are not an issue this is nothing critical that needs changing, just something that can be considered by the authors.

 The discussion section is helpful for drawing together the discussion and opens up the the door for future research to continue with similar studies either for other institutions or then more broadly in a quest to identify CPPs. Logical conclusions are made from findings of the study.

 A small correction/update I would suggest: In the summary an conclusions section you mention EQSIP in relation to the DIAMAS project. During the path of the project this has evolved into what is now called the The Diamond OA Standard (DOAS) for which there is a reference below, which might make more sense to refer to

 Consortium of the DIAMAS project. (2024). The Diamond OA Standard (DOAS) (1.2). Zenodo. https://doi.org/10.5281/zenodo.13820036

 So as you can see I have nothing that I ultimately require to be revised, moreso I want to mention a few questions related to CPPs and see if these could be relevant to consider in a revision of this study or perhaps as part of future endeavours:

 1. Could you perceive any "distancing" attempts at these journals from publishing works too dominantly by authors from their own institution? In universities I´ve noticed this persistent desire to create some kind of firewall for this type of activity not just related to publishing but more broadly in all areas, so it does not become an internal echo/merit accumulation chamber.

 2. From experience in researching this area myself I know that it is quite surprising how many publishing outlets are operated through different individuals and branches of a university, with seemingly no-one having a proper overview of everything going on in terms of supporting or coordinating the activities centrally. This perspective was not in my view fully explored in the interview excerpts given, the fragmented nature was more taken as a given. Did you gather any insight on the 1) desire of CPPs to get more central support, 2) willingness of anyone at the institution to look into this to explore support opportunities? 

 3. Related to the previous point still, from the manuscript it was interesting to read that few of the journals seemed to use any dedicated publication management software. Something like that could be quite nice for the university to provide for use should they approve of the publishing activity (many of the CPPs have “Cambridge” in their name). But I suppose it can become an issue of then becoming too close to the university so that publication practice standardisation, vetting of new titles, branding, communication and that kind of stuff becomes necessary to control which might suck the fun out of the volunteers active in these projects. Did you get any impressions or reflections on this point? It might seem like providing support for journals like this can carry risks of alienating the editors if done incorrectly.

 In all, want to congratulate the authors on a very high quality manuscript that taps into a phenomenon that easily is hidden in plain sight unless one makes a concerted effort to identify the parts of the whole, I have no reservations in suggesting this manuscript for acceptance pending that the authors take a look a the open questions I posed above and provide some responses in a slightly updated version of the manuscript if they have data to support such discussions.

Is the work clearly and accurately presented and does it cite the current literature?

Yes

If applicable, is the statistical analysis and its interpretation appropriate?

Yes

Are all the source data underlying the results available to ensure full reproducibility?

Partly

Is the study design appropriate and is the work technically sound?

Yes

Are the conclusions drawn adequately supported by the results?

Yes

Are sufficient details of methods and analysis provided to allow replication by others?

Yes

Reviewer Expertise:

scholarly publishing, open science, scientometrics, library and information science

I confirm that I have read this submission and believe that I have an appropriate level of expertise to confirm that it is of an acceptable scientific standard.

# Response

**Published:** 2025-07-16

**Authors:** Samuel Moore

**Affiliations:** Cambridge University Library, University of Cambridge, Cambridge, England, UK

**Thank you for the opportunity to review the paper ”“I just very much love the journal”: Understanding the  community-led publishing landscape at the University of  Cambridge”.**

**The study defines and conceptualises community-led publishing projects (CPPs) and conducts a mapping study on the CPPs active at Cambridge University, resulting in 34 CPPs, which is then followed up by qualitative interviews with 10 journal editors connected to the identified CPPs. The interview transcripts are inductively analysed to enable bottom-up identification of themes from the interviews. Over the years there have been many individual case studies written from the perspective of how individual community operated journals are surviving, often with scarce resources, but no study that would draw upon a set of journals that are often loosely coupled with a single institution exists to my knowledge, which in my view is a strong positive for the overall study design.**

**The different parts of the paper are sound and well-presented:**

**The introduction provides a good introduction into the topic area, which is related to the ongoing research and science policy efforts of diamond open access publishing, institutional publishing, and nonprofit publishing - all of which are relevant to the concept of CPPs that is introduced and defined very well. Resources are up to date and offer glimpses into the different topic areas that relate to this study.**

**The methods section is well-prepared and comprehensive, making it possible to fully follow the process that lies behind the paper and the analysis methodology for the interviews. A huge strength of the paper is that most of the interview transcripts are linked to as open data, so that it is possible to read them from start to finish if one would wish to do so, or incorporate them into future research.**

**The results presentation is solid and provides a generous amount of quotes extracted from the interviews to support the different identified themes and findings. If anything they could be slightly reduced or shortened to make the paper a bit snappier, some transcript segments are also quoted multiple times for example. Since the full transcripts are available for anyone wanting the complete narratives. In this publication format where print or word counts are not an issue this is nothing critical that needs changing, just something that can be considered by the authors.**

 We thank the reviewer for this comment. We have included several quotations to fully capture the discussions related to the themes as far as possible for individuals who may not wish to go through the full transcripts. Quotations are easily identifiable in the article and can be glossed over by the reader if need be.

**The discussion section is helpful for drawing together the discussion and opens up the door for future research to continue with similar studies either for other institutions or then more broadly in a quest to identify CPPs. Logical conclusions are made from findings of the study.**

**A small correction/update I would suggest: In the summary an conclusions section you mention EQSIP in relation to the DIAMAS project. During the path of the project this has evolved into what is now called the The Diamond OA Standard (DOAS) for which there is a reference below, which might make more sense to refer to**

**Consortium of the DIAMAS project. (2024). The Diamond OA Standard (DOAS) (1.2). Zenodo. https://doi.org/10.5281/zenodo.13820036**

 We thank the reviewer for this observation and have amended the reference.

**So as you can see I have nothing that I ultimately require to be revised, moreso I want to mention a few questions related to CPPs and see if these could be relevant to consider in a revision of this study or perhaps as part of future endeavours:**

**1. Could you perceive any "distancing" attempts at these journals from publishing works too dominantly by authors from their own institution? In universities I’ve noticed this persistent desire to create some kind of firewall for this type of activity not just related to publishing but more broadly in all areas, so it does not become an internal echo/merit accumulation chamber.**

 There was no real sense of this ‘distancing’ for CPPs at Cambridge. In fact, CPPs appear to be encouraged as alternative avenues for publication. However, we did not specifically ask questions to probe this and we acknowledge that it would be worth exploring this in future research.

**2. From experience in researching this area myself I know that it is quite surprising how many publishing outlets are operated through different individuals and branches of a university, with seemingly no-one having a proper overview of everything going on in terms of supporting or coordinating the activities centrally. This perspective was not in my view fully explored in the interview excerpts given, the fragmented nature was more taken as a given. Did you gather any insight on the 1) desire of CPPs to get more central support, 2) willingness of anyone at the institution to look into this to explore support opportunities?**

 We made the informed assumption that CPPs in Cambridge are fragmented and not centralised because of numerous reasons, including our experience working in this area at Cambridge, the lack of a central way to locate CPPs, and the generally siloed nature of initiatives within the University of Cambridge. This assumption was borne out in the data.

 During the interviews, we asked about the specific avenues of support CPPs needed, and these areas are explored in depth in the ‘Support’ theme and subtheme sections which detail the specific areas of support mentioned by interviewees. One of the areas of support was a more centralised system or, at the very least, a repository/database of CPPs currently operating at Cambridge. This research provides a snapshot of this information, insofar as it was possible to find CPPs at the time. One of the central goals of the research is to also identify what constitutes CPPs at Cambridge to then see whether the libraries or other university bodies can provide support in the future. We hope that because of this work, appropriate support can come to fruition, but the aim of this research was not to specifically find anyone/organisation willing to provide support opportunities as an outcome.

**3. Related to the previous point still, from the manuscript it was interesting to read that few of the journals seemed to use any dedicated publication management software. Something like that could be quite nice for the university to provide for use should they approve of the publishing activity (many of the CPPs have “Cambridge” in their name). But I suppose it can become an issue of then becoming too close to the university so that publication practice standardisation, vetting of new titles, branding, communication and that kind of stuff becomes necessary to control which might suck the fun out of the volunteers active in these projects. Did you get any impressions or reflections on this point? It might seem like providing support for journals like this can carry risks of alienating the editors if done incorrectly.**

 We did not directly explore this question in detail, but we agree that it is something that should be considered further.

**In all, want to congratulate the authors on a very high quality manuscript that taps into a phenomenon that easily is hidden in plain sight unless one makes a concerted effort to identify the parts of the whole, I have no reservations in suggesting this manuscript for acceptance pending that the authors take a look a the open questions I posed above and provide some responses in a slightly updated version of the manuscript if they have data to support such discussions.**

 We thank the reviewer for this comment.

# Reviewer report: Reviewer response for version 1

**DOI:** 10.5256/f1000research.177159.r375561

**Published:** 2025-04-29

**Authors:** Chun-Kai (Karl) Huang

**Affiliations:** 1 Curtin University, Bentley, Western Australia, Australia

This article explores the landscape of community-led publishing projects (CPPs). These are produced entirely by academics, librarians, and students, separating them from traditional commercial publishing. The authors identified 34 CPPs from a range of disciplines across the University of Cambridge, which were qualitatively explored for their practices, motivations, and other characteristics. This is supplemented with interviews of 10 participants from 7 journals.

 This is a timely contribution to the literature, as the world’s researchers, and related stakeholders, navigate through a changing academic landscape – where we seek for publishing models that can adapt to the changes and needs of the research community and beyond. Hence, a thorough evaluation of alternative models (like CPPs) from commercial ones is highly relevant in these ongoing discussions.

 The article is well-written, and the research design is sound. The authors have shown great care in both laying out the main findings from the research and in managing the interview data. The authors have made the data available where possible to support reproducibility.

 Below I list a few minor suggestions that the authors may wish to address further. They may not all be relevant depending on the data/information available – but may be useful to address one way or another.

- As the authors mentioned, a large percentage of the CPPs investigated were from the Arts, Humanities, and Social Sciences. Does that imply, if more CPPs from other disciplines were to be included, we might see significantly different results from the analysis? Or not? I appreciate that this research only provides a snapshot of limited CPPs from the University of Cambridge, and it may be difficult to address the question more generally. However, I wonder whether there are any signs of this from the current cohort?
- Sustainability, especially financial support, plays an important role for CPPs and are discussed at several places in the article. From the current data, is it possible to provide more granular analysis of how CPPs are funded – e.g., how many are funded by their departments, and how may receive external funding? In addition, are there indications of what the long-term financial sustainability would look like for CPPs – considering that some of these CPPs have been running for quite some time?
- This last point is also related to the previous two. While reading the article, I kept on wondering whether there could be a table listing characteristics contrasting some commonalities and differences between traditional commercial publishing and CPPs. I understand that the authors have specifically taken an expansive approach to what makes up a CPP – but listing some characteristics may help readers to have a clearer picture of what may be covered. Similarly, listing some examples of projects excluded from the study, and reasons for exclusion, may also be helpful.

Is the work clearly and accurately presented and does it cite the current literature?

Yes

If applicable, is the statistical analysis and its interpretation appropriate?

Yes

Are all the source data underlying the results available to ensure full reproducibility?

Partly

Is the study design appropriate and is the work technically sound?

Yes

Are the conclusions drawn adequately supported by the results?

Yes

Are sufficient details of methods and analysis provided to allow replication by others?

Yes

Reviewer Expertise:

Open Knowledge, Open Science, Open Access, Statistics, Data Science, Bibliometrics, Scientometrics, Probability Theory

I confirm that I have read this submission and believe that I have an appropriate level of expertise to confirm that it is of an acceptable scientific standard.

# Response

**Published:** 2025-07-16

**Authors:** Samuel Moore

**Affiliations:** Cambridge University Library, University of Cambridge, Cambridge, England, UK

**This article explores the landscape of community-led publishing projects (CPPs). These are produced entirely by academics, librarians, and students, separating them from traditional commercial publishing. The authors identified 34 CPPs from a range of disciplines across the University of Cambridge, which were qualitatively explored for their practices, motivations, and other characteristics. This is supplemented with interviews of 10 participants from 7 journals.**

**This is a timely contribution to the literature, as the world’s researchers, and related stakeholders, navigate through a changing academic landscape – where we seek for publishing models that can adapt to the changes and needs of the research community and beyond. Hence, a thorough evaluation of alternative models (like CPPs) from commercial ones is highly relevant in these ongoing discussions.**

**The article is well-written, and the research design is sound. The authors have shown great care in both laying out the main findings from the research and in managing the interview data. The authors have made the data available where possible to support reproducibility.**

**Below I list a few minor suggestions that the authors may wish to address further. They may not all be relevant depending on the data/information available – but may be useful to address one way or another.**

**As the authors mentioned, a large percentage of the CPPs investigated were from the Arts, Humanities, and Social Sciences. Does that imply, if more CPPs from other disciplines were to be included, we might see significantly different results from the analysis? Or not? I appreciate that this research only provides a snapshot of limited CPPs from the University of Cambridge, and it may be difficult to address the question more generally. However, I wonder whether there are any signs of this from the current cohort?**

 As noted above, we attempted to contact as many CPPs as possible without restricting ourselves to any particular disciplines. However, a significant portion of AHSS CPPs agreed to participate which was not in our control per se. It would be difficult to speculate whether or not there would be significant differences if CPPs from other disciplines were interviewed, but we do acknowledge that each CPP may have unique processes in their publication models but that there are overlaps which seem to be consistent across CPPs.

**Sustainability, especially financial support, plays an important role for CPPs and are discussed at several places in the article. From the current data, is it possible to provide more granular analysis of how CPPs are funded – e.g., how many are funded by their departments, and how may receive external funding? In addition, are there indications of what the long-term financial sustainability would look like for CPPs – considering that some of these CPPs have been running for quite some time?**

 Information about CPPs that were not interviewed was sourced from websites and it was almost never stated where and how they are funded. What we do know from the interviews is that funding is largely non-existent or limited to grants/funders and that most of the publishing work is conducted on a voluntary basis. Unfortunately, we do not have information about the specific ways in which CPPs are individually funded beyond what we have provided in the article. In Section 4, we provide a recommendation that future research explore the avenues of financial support of CPPs.

**This last point is also related to the previous two. While reading the article, I kept on wondering whether there could be a table listing characteristics contrasting some commonalities and differences between traditional commercial publishing and CPPs. I understand that the authors have specifically taken an expansive approach to what makes up a CPP – but listing some characteristics may help readers to have a clearer picture of what may be covered. Similarly, listing some examples of projects excluded from the study, and reasons for exclusion, may also be helpful.**

 As mentioned, we provide a clear definition of CPPs and a table distinguishing the differences between CPPs and professional publishers. Exclusion of projects can be inferred from what does and does not meet these criteria, where reasonable.

# Reviewer report: Reviewer response for version 1

**DOI:** 10.5256/f1000research.177159.r372906

**Published:** 2025-04-16

**Authors:** Abdelghani Maddi

**Affiliations:** 1 CNRS and Sorbonne University, Paris, France

This article investigates the underexplored world of community-led publishing projects (CPPs) at the University of Cambridge. Through a dual methodology combining a descriptive mapping of 34 active CPPs and qualitative interviews with 10 project stakeholders, the authors present an empirical study that illustrates the motivations, operational structures, challenges, and values associated with these non-commercial publishing efforts.

 The paper positions CPPs as an important yet fragile component of the scholarly publishing ecosystem, especially in the context of growing debates on Open Access (OA) and the need for alternative publishing models beyond the article processing charge (APC) framework.

 The article is timely, accessible, and relevant to both practitioners and researchers interested in scholarly communication, open science, and publishing reform. The authors demonstrate sincere commitment to the values of ethical research and open data. The interviews are well conducted and the voices of the participants are handled with care and respect. However, there are several conceptual and methodological weaknesses that limit the paper’s scholarly contribution, which I detail below.

 1. The research design combines a mapping exercise with semi-structured interviews. While this design is appropriate for an exploratory study, the execution lacks some transparency:

- The definition of a "community-led publishing project" remains vague. Without clear operational criteria, it is difficult to evaluate the inclusivity and boundary conditions of the selected 34 projects.
- The sample of 10 interviews — although rich in personal narratives — is small relative to the diversity the authors suggest exists within the Cambridge ecosystem. The paper does not discuss whether data saturation was reached, which makes the representativeness of the findings uncertain.
- The so-called "landscape mapping" is largely underdeveloped. Beyond the number “34”, no table, descriptive statistics, or classification are presented. This prevents the reader from understanding the variety and scope of CPPs at Cambridge.

2. Throughout the manuscript, the authors imply that certain characteristics — such as the reliance on unpaid labor, the diversity of content formats (articles, reviews, …), and the use of peer review without financial compensation — are inherent to CPPs. However, these features are widely shared across academic publishing as a whole, both in commercial and non-commercial contexts. The authors should revise the language to distinguish which findings are genuinely specific to CPPs, and which are reflections of broader academic publishing culture.

 3. The introduction suggests that the article aims to explore how CPPs could be supported "in the university and beyond." However, the concluding recommendations almost exclusively focus on institutional support mechanisms within universities, without offering any substantial consideration for extra-institutional avenues (such as public funding bodies, independent nonprofit infrastructures, …).

 The authors should clarify either:

- that their recommendations are intentionally focused on university-led support models,
  or
- expand the discussion to reflect the "beyond the university" dimension promised in the introduction.

4. The article would benefit from deeper engagement with relevant scholarly literature, particularly in relation to:

- the political economy of academic publishing;
- the ethics and structural precariousness of unpaid editorial labour in academic contexts;
- and the concept of “care” in scholarly communication, which is mentioned repeatedly but remains undefined and under-theorised.

To sum up, this article offers a timely and thoughtful contribution to the growing body of research on alternative scholarly indexing models. Its qualitative insights into the experience of community-led editors are authentic and relevant. With careful revisions addressing these clarifications and refinements, the article has the potential to become a well-grounded and valuable contribution to the field of scholarly communication.

**Data and ethical standards**

 The authors meet the ethical standards of F1000Research, particularly in relation to informed consent, anonymization, and open data availability. The transparency with which the authors handle sensitive qualitative data is exemplary.

Is the work clearly and accurately presented and does it cite the current literature?

Partly

If applicable, is the statistical analysis and its interpretation appropriate?

Yes

Are all the source data underlying the results available to ensure full reproducibility?

Yes

Is the study design appropriate and is the work technically sound?

Yes

Are the conclusions drawn adequately supported by the results?

Yes

Are sufficient details of methods and analysis provided to allow replication by others?

Yes

Reviewer Expertise:

Scholarly Communication, Scientometrics, Open Science, Research Evaluation, Research Integrity.

I confirm that I have read this submission and believe that I have an appropriate level of expertise to confirm that it is of an acceptable scientific standard, however I have significant reservations, as outlined above.

# Response

**Published:** 2025-07-16

**Authors:** Samuel Moore

**Affiliations:** Cambridge University Library, University of Cambridge, Cambridge, England, UK

**1. The research design combines a mapping exercise with semi-structured interviews. While this design is appropriate for an exploratory study, the execution lacks some transparency:**

- **The definition of a "community-led publishing project" remains vague. Without clear operational criteria, it is difficult to evaluate the inclusivity and boundary conditions of the selected 34 projects.**

We have added a clear definition of what we mean by community-led publishing projects. Specifically, we define them as “non-for-profit, locally run publishing initiatives that produce niche yet mostly academic content, with some connection to a university, in this case the University of Cambridge”. In addition, we provide a table describing some of the differences between CPPs and professional publishers (Table 1).

  

- **The sample of 10 interviews — although rich in personal narratives — is small relative to the diversity the authors suggest exists within the Cambridge ecosystem. The paper does not discuss whether data saturation was reached, which makes the representativeness of the findings uncertain.**

We did not formally reach saturation as a strategy for data collection because each CPP is quite different in many ways, but we attempted to contact as many CPPs as we could (at least 20) and carried out interviews with all who responded and agreed to participate.

  

- **The so-called "landscape mapping" is largely underdeveloped. Beyond the number “34”, no table, descriptive statistics, or classification are presented. This prevents the reader from understanding the variety and scope of CPPs at Cambridge.**

We provide a detailed table with detailed information of all 34 CPPs in the extended data, which is available on the repository ( https://doi.org/10.17863/CAM.111985). However, we are aware that this requires some work to access so we have moved the table into main article. See Table 2.

**2. Throughout the manuscript, the authors imply that certain characteristics — such as the reliance on unpaid labor, the diversity of content formats (articles, reviews, …), and the use of peer review without financial compensation — are inherent to CPPs. However, these features are widely shared across academic publishing as a whole, both in commercial and non-commercial contexts. The authors should revise the language to distinguish which findings are genuinely specific to CPPs, and which are reflections of broader academic publishing culture.**

 We have updated the paper to reflect this concern. However, the results presented are about CPPs but may also be relevant to other forms of publishing. We have clarified where this distinction is important.

**3. The introduction suggests that the article aims to explore how CPPs could be supported "in the university and beyond." However, the concluding recommendations almost exclusively focus on institutional support mechanisms within universities, without offering any substantial consideration for extra-institutional avenues (such as public funding bodies, independent nonprofit infrastructures, …).**

**The authors should clarify either:**

- **that their recommendations are intentionally focused on university-led support models,**
**or**
- **expand the discussion to reflect the "beyond the university" dimension promised in the introduction.**

We have amended the text to address this and have focused on universities in particular.

**4. The article would benefit from deeper engagement with relevant scholarly literature, particularly in relation to:**

- **the political economy of academic publishing.**
- **the ethics and structural precariousness of unpaid editorial labour in academic contexts.**
- **and the concept of “care” in scholarly communication, which is mentioned repeatedly but remains undefined and under-theorised.**

We thank the reviewer for this comment and have included a deeper discussion of these points throughout the article.

**To sum up, this article offers a timely and thoughtful contribution to the growing body of research on alternative scholarly indexing models. Its qualitative insights into the experience of community-led editors are authentic and relevant. With careful revisions addressing these clarifications and refinements, the article has the potential to become a well-grounded and valuable contribution to the field of scholarly communication.**

 We thank the reviewer for this comment.
